# Distribution of *Leptospira* Serogroups in Cattle Herds and Dogs in France

**DOI:** 10.4269/ajtmh.13-0416

**Published:** 2014-10-01

**Authors:** Florence C. Ayral, Dominique J. Bicout, Helena Pereira, Marc Artois, Angeli Kodjo

**Affiliations:** WildTech Project, VetAgro Sup, Marcy L'Etoile, France; Biomatématiques et Epidémiologie, EPSP - TIMC, VetAgro Sup, Marcy L'Etoile, France; WildTech Project, VetAgro Sup, Marcy L'Etoile, France; Laboratoire des Leptospires, VetAgro Sup, Marcy L'Etoile, France

## Abstract

A retrospective study was conducted to identify and describe the distribution pattern of *Leptospira* serogroups in domestic animals in France. The population consisted of cattle herds and dogs with clinically suspected leptospirosis that were tested at the “Laboratoire des Leptospires” between 2008 and 2011. The laboratory database was queried for records of cattle and dogs in which seroreactivity in *Leptospira* microagglutination tests was consistent with a recent or current infection, excluding vaccine serogroups in dogs. A total of 394 cattle herds and 232 dogs were diagnosed with clinical leptospirosis, and the results suggested infection by the *Leptospira* serogroup Australis in 43% and 63%, respectively; by the *Leptospira* serogroup Grippotyphosa in 17% and 9%, respectively; and by the *Leptospira* serogroup Sejroe in 33% and 6%, respectively. This inventory of infecting *Leptospira* serogroups revealed that current vaccines in France are not fully capable of preventing the clinical form of the disease.

Leptospirosis is a zoonotic bacterial disease that infects humans and domestic and wild mammals worldwide. This disease is important globally because of its worldwide distribution and its potentially fatal effects in humans. In Western Europe, France is one of the most affected countries, with a reported incidence of 0.37/100,000 inhabitants in 2011 (230 cases).^1^

The pathogenic agents of leptospirosis are bacteria from the genus *Leptospira*, specifically *Leptospira interrogans* sensu lato. Approximately 250 pathogenic serovars are now recognized and gathered into 24 antigenically related serogroups.^2^ Although *Leptospira* can be maintained in wet environments for weeks, the main source of the bacteria is a wide range of domestic and wild mammals carrying specific *Leptospira* serogroups. Rodents are the predominant maintenance hosts of the bacteria, whereas infected dogs and cattle are less prevalent as hosts but may pose an important public health risk. Indeed, infectious urine excreted by infected domestic mammals^3^ and its potential contact with human mucosa could lead to *Leptospira* transmission. In addition, leptospirosis induces significant economic losses caused by reproductive disorders in cattle herds. Vaccines against certain *Leptospira* serovars are available for humans, dogs, and cattle, but the range of *Leptospira* serogroups is much broader compared with the range that vaccination protects against. Additionally, there is no cross-immunity between *Leptospira* serogroups.

The vaccines available before 2012 for domestic animals in France only targeted dogs and included the serovars icterohaemorrhagiae and canicola. The canine vaccine has been augmented with the serovar grippotyphosa (Versican)^®^, and a bovine vaccine that includes the serovar hardjo is now available. Previous studies have questioned the reliability of these vaccines and have reported that certain uncommon serogroups are increasingly found to be the cause of clinical leptospirosis in the United States^4^ and Europe.^5^ Therefore, understanding the distribution of currently circulating serogroups is critical for prophylactic purposes and vaccine design. This study provides an overview of *Leptospira* serogroups in France that are currently circulating in dogs and cattle herds showing signs suggestive of leptospirosis.

From January 2008 to December 2011, veterinarians from across the country obtained samples from cattle and dogs showing clinical signs consistent with leptospirosis. Leptospirosis diagnosis was performed at the Laboratoire des Leptospires (Marcy L'Etoile, France) using a microagglutination test (MAT) as the reference test. The MAT was performed using a panel of antigens representing both ubiquitous serovars and locally prevalent serovars, with log2 dilution series between 1:40 and 1:5120 in dogs and between 1:50 and 1:6400 in cattle. The following *Leptospira* serogroups, with related serovars in parentheses, were tested in both species: Icterohemorrhagiae (icterohemorrhagiae, copenhageni), Australis (munchen, australis, Bratislava), Autumnalis (autumnalis, bim), Bataviae (bataviae), Grippotyphosa (grippotyphosa, vanderhoedoni), and Sejroe (sejroe, saxkoebing, hardjo, wolffi). Four additional *Leptospira* serogroups, Canicola (canicola), Panama (manama, mangus), Pomona (pomona, mozdok), and Pyrogenes (pyrogenes), were only tested in dogs.

For cattle, no consensus is reported on the titer cut-off required to define an infected individual. Previously, the MAT showed a sensitivity and a specificity of 95% and 90%, respectively, at a cut-off ≥ 1:50 compared with microbiological cultures.^6^ From this, occurrences of cattle leptospirosis at the herd level were determined by identifying signs suggestive of leptospirosis, such as reproductive disorders and the presence of at least one cow with MAT titers ≥ 1:400. The cut-off was arbitrarily defined to increase the previously mentioned specificity. The predominant serogroup was then defined based on the maximum titer directed against one serogroup.^7^ Cross-reactivity between serogroups frequently occurs in MAT^8^ and results from a lack of specificity, especially from predominant non-specific immunoglobulin M (IgM) antibodies at the onset of infection.^9^ In these cases, MAT results involve maximum titers directed against two or more serogroups, thus preventing determination of the infecting serogroup. The MAT results including maximum titers directed against two serogroups are still informative by indicating one or the other as potentially circulating. In contrast, consideration of more than two possible circulating serogroups is speculative and uninformative. Therefore, among MAT results including maximum titers directed against two or more serogroups only the ones with two serogroups (“mix” results) were considered in this study.

Most dogs monitored by veterinarians are vaccinated, which results in the development of seroreactivity directed against the icterohaemorrhagiae, copenhageni, and canicola serovars (called vaccine serovars). As previously described, in the current study, occurrences of canine leptospirosis were defined by signs suggestive of leptospirosis, such as acute renal failure or liver failure and at least one MAT titer of ≥ 1:640 against non-vaccine serovars.^10^ The predominant serogroup was defined similar to that in cattle. However, the MAT results for which vaccine serogroup titers were equal to non-vaccine titers were analyzed separately, to minimize the impact of vaccination on the results.

To assess potential variation in the serogroup spread, mainland France was divided into six areas: North, Northwest, Northeast, Central, Southwest, and Southeast. The determination of the location of the different animals tested for the serogroups was based on the owner's address.

The MAT results of 394 cattle herds (570 cattle) suspected of having leptospirosis were used to determine the distribution of serogroups circulating in France. These MAT titer results ranged from 1:400 to 1:6400, with a median of 1:800. The predominant serogroups were Australis, Sejroe, and Grippotyphosa, regardless of the titer cut-off (Figure 1), and a similar serogroup ranking was observed in the six defined regions (Figure 2). In total, 29 herds were found to contain cows with MAT results suggesting different predominant serogroups. The combinations of Australis and Sejroe (*N* = 16) and of Sejroe and Grippotyphosa (*N* = 7) within a herd were predominant.

**Figure 1.**
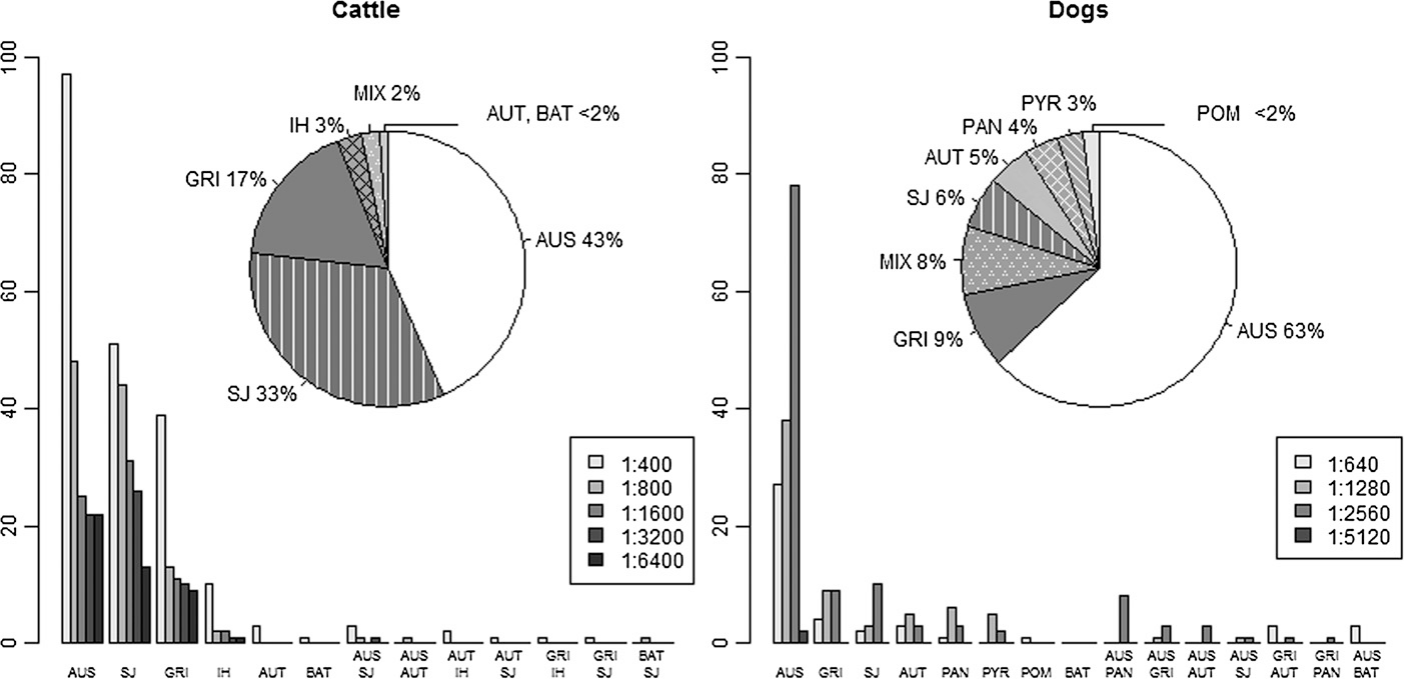
The distribution of *Leptospira* serogroups among 394 cattle herds and 232 dogs with suspected clinical leptospirosis (excluding microagglutination test (MAT) results indicating high icterohaemorrhagiae, copenhageni, and canicola titers in dogs). The bar plots show the distribution of the serogroups among 570 cows and 232 dogs, considering the maximum MAT titer. Australis (AUS), Autumnalis (AUT), Bataviae (BAT), Grippotyphosa (GRI), Icterohaemorrhagiae (IH), Panama (PAN), Pomona (POM), Pyrogenes (PYR), and Sejroe (SJ), results including maximum titers directed against two serogroups (MIX).

**Figure 2.**
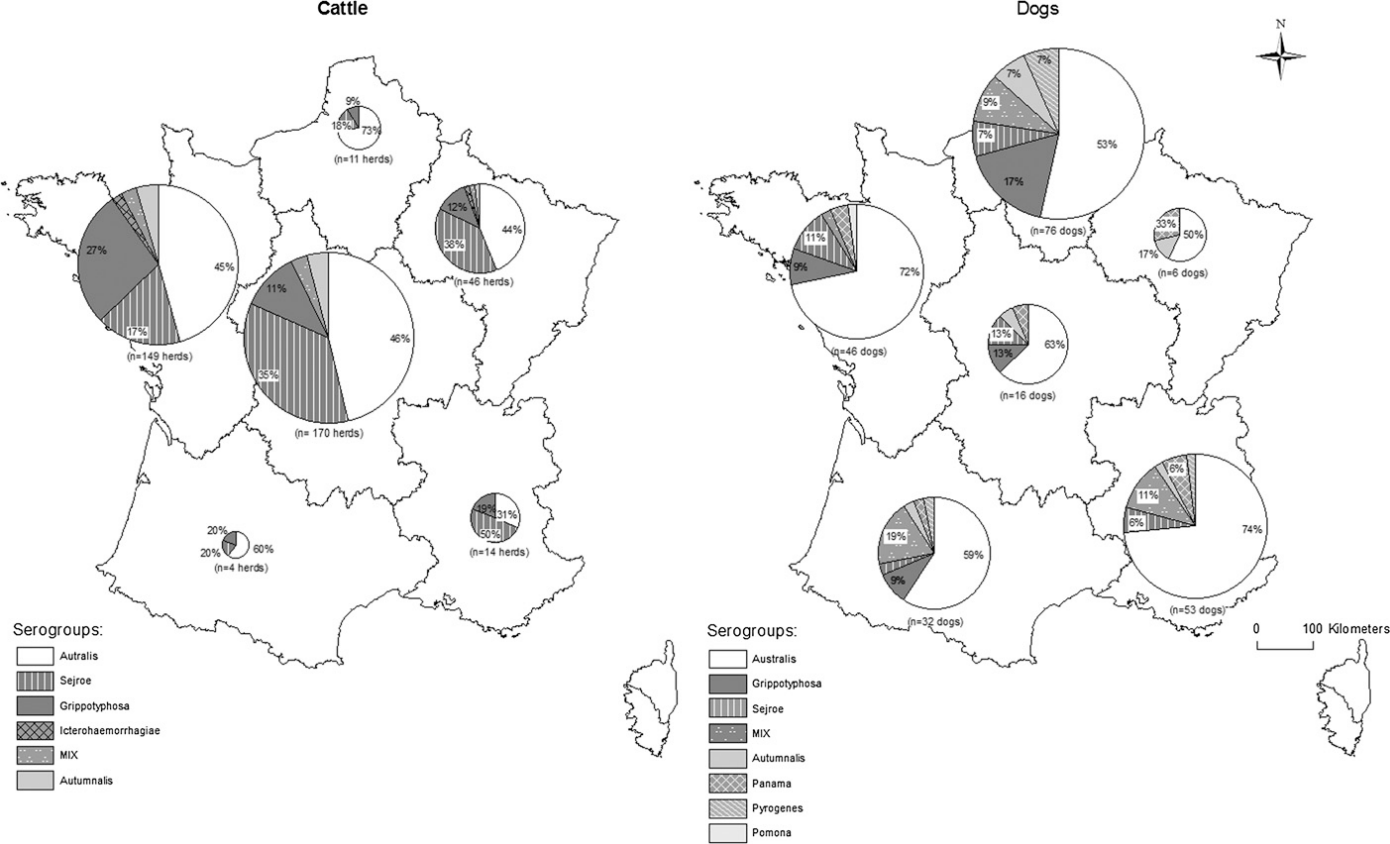
The spatial distribution of the infecting serogroups in 394 cattle herds and 229 dogs in six areas in mainland France: North, Northwest, Northeast, Central, Southwest, and Southeast. Three dogs were not considered because of missing location data, and one dog from Corse was excluded.

The MAT results of 232 dogs were included in the distribution. The MAT titer results ranged between 1:640 and 1:5120, with a median of 1:2580. According to the bar plots, the predominant serogroups were Australis and Grippotyphosa, regardless of the titer cut-off. In particular, Australis was predominant in all six regions, whereas Grippotyphosa was only recorded in the four regions of western France (North, Northwest, Central, and Southwest).

In all, 66 dogs with equal maximum titers directed against vaccine serogroups and one non-vaccine serogroup were additionally considered. The distribution of the non-vaccine serogroups was Australis (75%), Pyrogenes (14%), Grippotyphosa (5%), Sejroe (5%), and Panama (< 2%).

This study examined the distribution of infecting serogroups involved in clinical bovine and canine leptospirosis. The serogroups Australis and Grippotyphosa were consistently predominant in the two species, and the results in dogs were consistent with the findings of a previous study in Germany.^5^ Considering the sensitivity (Se) estimates related to the cut-off defined in dogs (Se = 22–67%),^10^ the occurrence of leptospirosis may have been underestimated in this species. Nevertheless, the specificity (Sp) estimates in cattle (Sp ≥ 90%) and dogs (Sp = 69–100%) and the high median titers associated with signs suggestive of leptospirosis supported a diagnosis of current or recent *Leptospira* infection. As previously reported, the MAT correctly predicted the infecting serogroup in 46–86% of cases^7^,^11^; the presumptive serogroup data appears to provide a broad overview of the serogroups commonly present in a population. Specifically, the majority of results (> 60% in cattle and > 72% in dogs) suggesting Australis and Grippotyphosa infections and the consistency of the distribution, regardless of the cut-offs used for the two species, provided substantial evidence for Australis and Grippotyphosa predominance in bovine and canine leptospirosis. These results also suggested that dogs and cattle could be exposed to the same sources of infection and/or the same infection pressure.

The spatial distribution of the predominant serogroups in cattle appeared homogeneous in all six regions, whereas in dogs, the distribution of Grippotyphosa was heterogeneous. This finding suggested that in contrast to cattle, the exposure of dogs to certain serogroups varied within mainland France.

The results of this study indicated that Sejroe was responsible for 30% of cases of bovine clinical leptospirosis. This finding suggested that the available bovine vaccine targeting this serogroup is capable of preventing one-third of the clinical cases. Nevertheless, additional serogroups, such as Australis and, to a lesser extent, Grippotyphosa, should be included to eliminate most *Leptospira*-related diseases in cattle. For dogs, it would be important to include Australis antigens in a canine vaccine to aid in preventing infection with the serogroup responsible for most clinical cases of leptospirosis in dogs in France.

This inventory of infecting *Leptospira* serogroups circulating in cattle and dogs should be considered when designing future vaccines to improve leptospirosis prevention. As part of a “one health” approach, this could lead to reduce human cases exposed to potentially infected domestic animals.
